# Supplementary material for: “Why take the patient back home?”: Exploring the lived experiences of caregivers of COVID-19-infected individuals in Blantyre, Malawi
Source: PLOS Glob Public Health. 2023 Sep 27;3(9):e0001601. doi: 10.1371/journal.pgph.0001601 (PMC10529612; doi:10.1371/journal.pgph.0001601)
Supplement: S2 File — (DOCX) [file pgph.0001601.s002.docx]

**Introduction for Participant**

Good day. My name is ………………………. *(your name)* and I work as a research assistant at the College of Medicine. You ………………………. *(name of the participant)* have been identified as a key individual who can provide information, opinions and perspectives on the corona virus (COVID-19).

***INTERVIEWER: PLEASE ADMINISTER THE CONSENT FORM***

Please indicate whether you are willing to participate in the study by answering the following questions.

1. Do you understand that no identifying information about you will be shared in our final report?

Yes  No

1. Do you understand that you can choose not to answer any questions that you feel uncomfortable to answer?

Yes  No

Thank you so much for agreeing to have this interview with me. Before we start, I would like to know more about you.

1. **Demographic Data**

Date of birth :____/____/_____ (DD/MM/YYYY)

- *If not known*, please provide estimated age :   years

Marital Status : ___________

- Has the participant’s marital status changed over the past 2 years? Yes No
- *If yes*, please specify:________________

Highest Educational Attainment : ___________

Current Occupation : ___________

- Has the participant’s occupation changed over the past 2 years? Yes No
- *If yes*, please specify

Name of area of Residence: ___________

For how long have you been living in this area?:  days/weeks/months/years *(tick applicable)*

- Gender: Male Female

**Section A- General knowledge on COVID-19**

1. Describe in details what you know about COVID-19.
2. Describe the sources of your information.

**Section B- Lived experience of caregivers**

1. Explain in detail how you learnt about your relations’ COVID-19 infection.
   1. Probe on symptoms prior to testing
   2. Decision making processes: what did they think about the symptoms?/ what did they do? Why did they do that? Who did they consult? / What made them to go for testing or refuse?
   3. Probe on the process of getting the diagnosis/ What did they think or how did they react when they heard the diagnosis?
   4. Probe on the process of informing family members/ Who did they speak to first? Why? What were their reactions?
2. Explain in detail what challenges you have experienced caring for your patients
3. Probe on health care
4. Probe on information needs/deficiencies
5. Probe on daily welfare
6. Managing their domestic roles, work obligations, business & other roles at church/community etc
7. Probe on isolation-what did they do to self-isolate? What worked well? What factors facilitated or impeded compliance to self-isolation/ travel restrictions? What did you think about the benefits & risks of all these preventive measures to you as an individual? How about the community at large?
8. Probe on stigma and discrimination-How did family members/ neighbors/ workmates/ other social groups e.g church support you during the illness and after the illness? Explain any challenges you experienced?
9. Explain in detail how you have tried to overcome the challenges you have experienced in providing care to your patient
10. Probe on health care
11. Probe on information needs/ deficiencies
12. Probe on daily welfare
13. Probe on isolation
14. Probe on stigma and discrimination
15. Explain in detail how government should be assisting people like you who are providing care to your patient
16. Probe on health care
17. Probe on information needs/deficiencies
18. Probe on daily welfare
19. Probe on isolation
20. Probe on stigma and discrimination
21. Explain in detail how community members should be assisting people like you who are providing care to your patient
22. Probe on health care
23. Probe on information needs/deficiencies
24. Probe on daily welfare
25. Probe on isolation
26. Probe on stigma and discrimination

**Closing Remarks**

We are now at the end of the interview, is there anything you would like to add on COVID-19?

Do you have any questions for me?

Thank you very much for your time.
