# Supplementary material for: “Why take the patient back home?”: Exploring the lived experiences of caregivers of COVID-19-infected individuals in Blantyre, Malawi
Source: PLOS Glob Public Health. 2023 Sep 27;3(9):e0001601. doi: 10.1371/journal.pgph.0001601 (PMC10529612; doi:10.1371/journal.pgph.0001601)
Supplement: Attachment — Submitted filename: Letter of Response.docx [file pgph.0001601.s003.docx]

Dear Editor and Reviewers,

Thank you for reviewing our manuscript which has improved the presentation and quality of it. Please find below our responses to the queries raised by the reviewers.

Sincerely,

Alinane Linda Nyondo-Mipando (Corresponding Author)

Reviewer #1: In this manuscript, the authors describe the experiences of covid-19 caregivers in and around an urban centre in Malawi. The authors have fairly described the previous literature on the topic, contextualizing their results. Thank you to the authors for this thoughtful manuscript which describes important experiences for which there has been little research.

Major Revisions Suggested
1. Overall the methods are well described, but there are 2 areas which need strengthening. Firstly, the analysis method was described as thematic, but appeared to be a deductive analysis based on the theory of caregiver dynamics. The "themes" seemed to be entirely consistent with the concepts in the theory. This is also reflected in Figure 1 which does not seems to contain little study-specific information, but mostly just represent the theory. I suggest: 1) introducing the theory at an earlier point in the methods section and giving it its own heading,

**Response: Thank you for this comment. We have revised and have included a separate section for the theory before the analysis part in the methods section as follows:**

**Theory for Analysis: Theory of caregiving dynamics**

**The qualitative data on the lived experiences of the caregivers were analysed and categorised following a deductive approach and guided by the theory of caregiving dynamics [15]. This theory shows how the caregiving role changes and grows over time and has three constructs as follows: commitment, expectation management, and role negotiation. Commitment entails that a caregiver provides care to the patient irrespective of the risks, challenges, and lack of self-prioritisation that comes with the role.  Expectation management states that a caregiver and a patient have expectations such as worsening or a better prognosis of the condition of the patient and includes the roles that both a caregiver and patient have to fulfill. Expectation management consists of envisioning tomorrow, getting back to normal, taking one day at a time, gauging behavior, and reconciling treatment twists and turns.  Role negotiation entails a caregiver pushing and getting grips with the caring responsibility, sharing responsibilities, attending to or listening to the patient’s voice, and vigilant bridging through negotiations with the health system in the care of the patient.**

2) calling this a deductive analysis,

**Response: We agree with the reviewer that this was more of a deductive approach with themes deduced from the theory. We have acknowledged that we added some elements in the theory from the data as appropriate.**

2) having 2 figures in the paper, one which represents the theory (with no data) and the other figure which displays how the data from study supports the theory.

Response: This is noted. Figure 1 below illustrates the original figure while Figure 2 shows how the researchers fitted their results to illustrate the findings under each construct of the theory. Our illustration adds a family’s economic status as a factor that largely influenced the caregiving dynamics.

IllnessTrajectory

Commitment, Self care

Expectation Management,New Insight

Role Negotiation, Role Support

Future Caregiving Relationship

Present Caregiving Relationship

Past Caregiving Relationship

Figure 1- Caregiving dynamics

Role Negotiation: Getting the handle of the role while sharing responsibilities.

Role Support: receiving helpful information, finding support, meeting financial responsibilities

Lived experiences of Caregivers of COVID-19 patients in Blantyre Malawi

Commitment:

Enduring responsibility and Home management:

Making the patient a priority

Implementation of preventive measures-Self-Care

Expectation Management:

Envisioning Tomorrow and spiritual reliance

Economic Influence on the Management of a COVID-19 patient at home

Availability, non-availability or loss of income, maintenance of economic resources

Figure 2- Authors’ Adaptation of the Theory of Caregiving Dynamics

2. There is very little information in the manuscript on the Malawian health care and home care guidelines for those with covid-19. It would have been very useful to know if there were any guidelines on what kinds of patients were admitted to hospital (versus sent home with caregivers), the quarantine guidelines (if any) for household contacts of covid-19 patients, government recommendations (if any) for in-home distancing, cleaning, masking, etc. A fuller description of these items would strengthen the manuscript.

**Response: The following information on the guidelines and management that Malawi developed and implementd have been added to the background**

**Hospital admission is reserved for severe cases with a critical disease, severe respiratory distress or hypoxemia, aggravation of co-morbidities, shock, and severe bacterial or viral co-infections among others ( COVID-19 case management manual, June 2021) [4]. In contrast, the mild and moderate cases of COVID-19 are managed at home, focusing on preventing transmission to others through isolation from family, pets, and the public, strict physical distancing, hand hygiene, cough etiquette, cleaning, and disinfection of frequently touched surfaces and monitoring for deterioration.**

**The Malawi Ministry of Health national guidelines for quarantine recommends that a person who had close contact with a confirmed case of COVID-19 within a period of 0 to 14 days or a traveller entering the country from other countries affected by the COVID-19 pandemic to be quarantined for 14 days. Home quarantine was described as the voluntary isolation of an exposed person in the home that meet several criteria, including a fence separating the suspect from the rest of the family or community, dedicated room beddings, towels, cutlery, cups, and plates only for their use, to last them for the duration of the 14 days. The rooms that the exposed person use should be well-ventilated and preferably self-contained, among other criteria, and if some of the criteria are not met, the guidelines recommend institutional quarantine. Ideal sites for quarantine are the hotel, school dormitories, institutional hostels, and other facilities that appear appropriate to cater to groups of people. During the quarantine, the recommendation is to avoid sharing, eating, sleeping in different spaces, and staying at least 4 m away when passing or in shared toilet facilities if applied. The government was obliged to orient and provide educational material to persons in the home of the exposed persons to ensure the understanding of IPC**

3. A key underlying theme of the study is that the family's economic resources influenced all aspects of caregiving. This was displayed as a central/fundamental concept in Figure 1, but explicitly acknowledged as a key concept in the written results. Adding a sentence describing how this concept undergirded the others would be more clear.

**Response: We have included the following statement to clarify the aspect of a family’s economic resources greatly influencing the caregiving role.**

**Participants narrated that their ability to live in a home where isolation was possibly hinged on their finances. The economic level of a family influenced the commitment level of a caregiver through their availability to forego income-generating activities to provide care. Furthermore, it influenced their expectations of the future and others considered financial aspects. Economic levels also influenced the navigation of their role as a caregiver and required them to depend on others to meet their financial demands if a family has such links and if not then their role was negatively affected because they had low levels of income to execute their role effectively.**

Given the centrality of this concept, there needs to be more information in the methods and results regarding the economic status of the participants.

**Response: Our setting described the economic landscape of Blantyre. We have also acknowledged the limitation in our study to tease out more of the measure in ascertaining economic levels. Our results only offer the area of residence and occupation factors illustrating the economic status. The limitations are as follows: Future studies should tease out the baseline economic activities in detail so that they challenge or support the assertions in this study**

The authors frequently mention the impact of caregiving on poor and ultra-poor households, but we do not know how many caregivers from these households participated in the study (neighborhood was used as a proxy, but is not particularly descriptive). How were covid-19 cases recruited to the main study? Could this have influenced the SES of the caregivers? What kind of variation was Participants must have had a access to a phone to participate, meaning that they may not have been "ultra-poor".

**Response: We have added more details to the recruitment of our participants. When we noted that we had fewer participants recruited from rural areas, we increased our sample size to specifically recruit from that area so that we have a good representation of such participants. This was a challenge that was embedded in the methods we employed. We sampled our participants from those that enrolled in the epidemiological study as such our pattern mimicked that of the mother study. Of note, most COVID-19 patients in Malawi were from the urban areas and not the rural areas, however, our urban areas have variations in economic status. We did not consider access to a phone as a requirement. Ownership of a phone is 36% and 52% among women and men respectively in the southern region of Malawi as of 2019, 45% vs 49% in Blantyre rural and 71% v 71% in urban Blantyre. The added details are as follows:**

**We considered variations in marital status, education level, location of residence, and gender.**

**The study was inclusive of caregivers above the age of 18 years, willing to participate, and the primary caregiver of a COVID-19 patient enrolled in the main study and we considered having participants with variations in characteristics.  Thirty-seven caregivers were deemed adequate because Guest et.al., [11] argue that by the 12th interview, one would have captured 97% of the required information. We further observed similar codes in subsequent transcripts of the urban-based caregivers with few transcripts for rural based-caregivers which was also in keeping with the pattern of the pandemic, with the urban areas worst affected than rural settings. We, therefore, increased the sample size for the rural-based caregivers to check if they would be different meanings in the codes that were generated among the rural-based caregivers to maximise the number of rural-based caregivers as they were few. We noticed no new information suggesting different meanings for the generated codes as we progressed with the additional interviews suggesting we had reached data saturation (Hennink et., al 2022, Francis et.,al 2010).**

Also 27 participants were college educated, which may indicate higher economic status. Also there is a suggestion that participants were compensated for their interview, but there is no mention of the amount, which may have influenced participation. These methodological consideration and thier impact on the results should be further discussed as a limitation to the study (if in fact it was a limitation).

**Response: We have included the compensation that was made and it was one of the reasons for the lack of participation because some considered it low. We have acknowledged the limitations as follows:**

**Our sample had more participants who had a college-level education which could make the results more applicable to such a group. We deliberately recruited more participants to ensure that we have an equal representation of those who dwelled in the rural areas, which was a challenge because there were not as many as in the other areas. Future studies should tease out the baseline economic activities in detail so that they challenge or support the assertions in this study and could consider limiting the study to rural areas alone and conducting a case study.**

Minor Revisions Suggested

1. Justification of sample size and cessation of data collection requires more support. Were concepts fully developed? Why did the AUTHORS (who are familiar with the setting and the topic being studied) expect that the sample size would be adequate?

**Response: Our sample size was guided by assertions by Guest that state that by the 12^th^ interview one would have reached 97% saturation of ideas. We have included more information on sampling and saturation as follows:**

**Thirty-seven caregivers were deemed adequate because Guest et.al., [11] argue that by the 12th interview, one would have captured 97% of the required information. We further observed similar codes in subsequent transcripts of the urban-based caregivers with few transcripts for rural based-caregivers which was also in keeping with the pattern of the pandemic, with the urban areas worst affected than rural settings. We, therefore, increased the sample size for the rural-based caregivers to check if they would be different meanings in the codes that were generated among the rural-based caregivers to maximise the number of rural-based caregivers as they were few. We noticed no new information suggesting different meanings for the generated codes as we progressed with the additional interviews suggesting we had reached data saturation (Hennink et., al 2022, Francis et.,al 2010).**

2. The interview guide was not described and the supplemental file 2 was not present. The text should be able to stand alone and should contain a description of the interview guide and how it was developed? Was the theory of caregiver dynamics also used to develop the interview guide?

**Response: We have included the interview guide as a supplementary document and have also provided the main questions in the manuscript as follows:**

**The broad section of the guide inquired about their lived experiences and has subsections on challenges faced, solutions, and support received as they cared for their patient.**

**We did not use the theory of caregiving dynamics when we prepared the interview guides.**

3. Criteria used for purposive sampling should be described

**Response: We have included more information on the purposive sampling approach as follows:**

**We considered variations in marital status, education level, location of residence, and gender.**

**The study was inclusive of caregivers above the age of 18 years, willing to participate, and the primary caregiver of a COVID-19 patient enrolled in the main study and we considered having participants with variations in characteristics.** 

4. More information on the process of transcription and translation is required. Was any verification of transcription and translation completed? Was the transcript verbatim?

**Response: All data that were in Chichewa were translated into English by a transcriber who is fluent in both languages. LSK verified the translated transcripts**

5. More information was needed on the "coder". Coding is a key component of qualitative research and the training of this person and how the research team worked with this person was not sufficiently described.

**Response: Once the codebook was ready, all data was coded by an independent data coder, a social scientist who is trained and fluent coder, conversant with NVivo who was oriented to the codebook and was available for discussions as the coding process progressed. The coder held iterative meetings with the researchers to discuss the inclusion of more codes in the codebook or a combination of overlapping codes. The coded data were reviewed for completion and accuracy by MC and ALNM.**

6. Another 1- 2 sentences on how the researchers introduced themselves to the participants and the study prior to informed consent would be helpful.

**Response: The researchers identified themselves as research assistants in the SCATHIM study taking place at Kamuzu University of Health Sciences and explained that it was a study that recruited COVID-19-infected patients.**

7. The heading scheme is the results section is confusing. I would encourage the authors to explicitly identify the number of major and minor themes and then revise the heading scheme to make this more clear.

**Response: The headings have been revised and we have outlined the four main themes in our results these are: Economic influence on the management of a COVID-19 patient at home, Commitment, Expectation Management, and Role of a Caregiver. The subthemes under each theme have been named as such so that it is clear that it is a subtheme. Our Figure 1 has been updated to reflect the labelling.**

8. In the results section, I suggest using distinct terms to differentiate the practice of caregivers remaining in the home (to prevent transmission of covid-19 to non household members) and family members keeping to separate parts of the home (to prevent transmission of covid-19 to other household members). I believe the authors used the word isolation for both and this was somewhat confusing

**Response: Thank you for this comment, we have clarified that the isolation that is in discussion is among household members and not the non-household. This section is limited to the household and not the other non-household members. In the successive parts of the results, we have used prevention of transmission to others as opposed to isolation so that the distinction is clear.**

9. In the results, several quotes appear disconnected from the sentence immediately preceding the quote. For example on the bottom of page 19 (older children assume household tasks) and the middle of page 21 (people in social networks gave advice on how to care for covid-19 patients). I suggest to revise the results so there is a very tight connection between the idea expressed just before the quote and the contents of the quote. This will strengthen the credibility of your results (and conclusions).

**Response: Thank you for this comment. We have decided to delete that part as it was not fitting well especially with the quote while the rest of the narrative was in sync with the quote.**

**The narrative on social networks has been revised and it now reads:**

**In the absence of concrete information platforms, caregivers sought information from their social networks. Participants stated that the lack of readily available information due to the novelty of the condition forced them to solicit information from those within their social circles and these included healthcare workers and other members of the community.**

I also suggest that you include a quote which more clearly expresses the idea that the caregiver's future expectations were challenged by the high case fatality rate associated with covid-19. The data supporting this claim (which was mentioned in the abstract and the discussion) is not strong.

**Response: We have included the following quotes:**

***“I was heartbroken and very disappointed with the news because of the way COVID-19 is killing people out there. So I knew that if I will be very disappointed, then I will make the patient very disappointed and worried too… I was very heartbroken when I first hear about the results.” Participant Blantyre Rural SCA 1012, Blantyre Rural Caregiver***

***“I was filled with fear since we hear rumors every day about people dying, but still, I tried to conceal my fear even though I was afraid too. I asked myself: what will happen next?” SCA 1015 Blantyre High-Density Urban Caregiver***

10. In the abstract, the second sentence about WHO guidelines does not add to the abstract and could be deleted.

**Response: The sentence has been deleted.**

Reviewer #2: Topic: Why take the patient back home?”:exploring the lived experiences of caregivers of COVID-19 infected individuals in Blantyre, Malawi.

This is an important paper for Malawi given the guidelines on managing non severe cases of COVID 19 at home. It can support improvements in this area to help caregivers cope better with more standard information and support.

**Response: Thank you for this comment. We agree that the results will help in shaping the home management of COVID-19 and other infectious diseases.**

Abstract:
It is well written with a clear summary of the study.

**Response: Thank you for this response**

Introduction:
Capitalise the I in the introduction section heading.

**Response: Thank you for noting this and it has been corrected**

Generally, well written with studies in other settings reviewed to provide context to this paper.

**Response: Thank you for this comment**

Methods

1st paragraph. There two statements that are repeated. “Most residents are informally employed or run small-scale, informal businesses as a source of income…” You need to delete one. Either the 1st or the one coming sentences later.

**Response: Thank you for noting this repetition. We have corrected the sentence.**
 
Four seasoned qualitative research assistants, three female and one male namely MC, WN, LC, and RS conducted the digitally recorded telephonic
interviews. - Are these co-authors? Some initials are not anywhere in the author list.

**Response: We have removed the initials as they are not part of the authors.**

How much was the compensation? This is not provided, and yet one of the reasons for declining to participate was a low compensation amount. Please state clearly how much it was. May be under ethical considerations?

**Response: This has now been provided under data collection and it was MWK2000**

13 refusals out of this sample size approached is a substantial number. It is a very high refusal rate. It is also possible that those who declined because of compensation not being enough are of a particular social economic status. Could this introduce any bias to the study? Selection bias may be? Could this be acknowledged? It could also be because these were phone interviews in a setting where people value face to face interviews. No doubt, one reason was being uncertain of interviewer’s identity

**Response: The refusal rate was quite high and we have acknowledged it as a limitation of this study owing to the nature of the condition under study and the approaches used in data collection.**

Page 9, 1st sentence. Twenty-four caregivers were employed and 15 resided in low density urban areas of Blantyre, respectively. - What does respectively mean in this sentence?

**Response: The word respectively has been deleted.**

Page 10, last sentence. “Figure 1 below illustrates the interconnection of the factors with economic factors as the platform of the caregiving experience. - Take out the “below” because you do not determine where the figure will be located in the final paper. A figure reference is all you need I think. The figure may be location 2 pages away from this sentence for example, in the final paper.

**Results: Thank you for this insight and the word below has been deleted**

This comment may mean a modest revisit of the results. Although I am not proposing quantifying qualitative results, it helps to put some magnitude in the findings. How many participants mentioned somethings may be important to gauge how important that result is. It is not clear to know from the results currently if everything mentioned was coming from majority of participants, minority or even deviant cases or just one. Also providing for the categorization in the findings as it is in the sample would be important. Are some issues more pertinent for rural participants, or urban for example? This I believe is vital. Just providing two quotes, one from urban and another rural does not make the submission necessarily relevant to both. It could be just that one participant quoted from urban out of 15 that said something.

**Response: Thank you for this comment, we have reviewed the results and have highlighted whether it was commonly stated. We have refrained from counting and showing frequencies because it will not be consistent with the way we designed the study and analysis. We have also indicated if it was mostly stated by rural or urban dwellers. To ensure that the methodological challenges inherent in a qualitative inquiry are taken care of, we have recommended that future studies may consider a triangulation of methods so that the findings are richer and more comprehensive.**

Page 12: On the other hand, financially stable caregivers especially those that were formally employed stated that managing a COVID-19 patient at home, enabled them to save because they never spent money on transportation.
“On my part, I saved on fuel because I am not that mobile, I am not going
to any drinking joint, … I cannot say that it impacted me negatively, but it
did on my savings, so my savings were huge.” Participant 1007 High-
Density Urban Caregiver

This quotation does not seem to bring out the explanation that is synthesized. The participant says they are not that mobile anyway. So as a a caregiver, if they were not mobile from prior, how is this changing when they have someone to care for at home? I don’t see how the quote relates directly to enabling the participants save. Is there a better quotation, since this is a theme anyway? I believe you have several.
Response: This quote has been removed as it did not fit.
Page 13: Caregivers prioritised the family's health over other financial demands like bills and rentals and ensured that they had finances to cater to their medical-related bills. - What is a Bill? You mention bills here twice. What did you mean by the 1st bills stated, because medical costs are also bills.

**Results: We have clarified the meaning of bills and have specified them as groceries and medical costs.**

Page 14: Some caregivers said that despite being positive for COVID-19 as well, they prioritised the care of the index patient over their illness because of the severity of the illness of their patient. - what do the authors mean by the severity of the illness here? It is clear that all severe cases are managed at health facilities, as stated in the introduction. Now they introduce two points here. The index patient and severity. You need to explain what was meant clearly. If the index patient was not as severe as the other patients, what would happen? If the patient was severe, were they managed at home?

**Response: Thank you for this comment, it has been clarified as follows:**

**A minority of the caregivers who disclosed being infected with COVID-19 as well stated that t despite being positive, they prioritised the care of the patient over their illness because they were asymptomatic while their patient was symptomatic and sick and needed to be cared for. Others stated that they continued taking the same medications their patient was taking and followed the same supportive care like steaming, and use of herbs as was prevalent at the time.**

There are several areas where the authors just provide a one line result and then quotation(s). They need to synthesise the results and provide some detail from the data.
For example on page 17: Families changed the way they related emotionally to one another as a means of minimising the transmission of the virus. - It is not clear how they were emotionally relating before, and how the caregiving changed that relationship. Only providing a quote from one participant does not explain this.

**Response: We have revised the results and have included more information in the narratives where there was very minimal information.**

**Measures of isolation were extended to the conduct within a relationship. Participants that were used to relating emotionally through hugs before the infection, had to change their conduct. Families changed the way they related emotionally to one another as a means of minimising the transmission of the virus.**

Page 19: Some caregivers depended on their faith as a coping mechanism, especially in instances where the family members remained COVID-19 negative while nursing a positive patient in the home. – I don’t see how this is a coping mechanism. The quotes provided only show what happened after. They do not indicate that during the care process they relied on their faith. May be it is the writing that needs modification.

**Response: This has been revised as follows:**

**Some caregivers that belonged to a church and were spiritual depended on their faith as a coping mechanism. This was common among households where the family members remained COVID-19 negative while nursing a positive patient in the home. In such households, participants believed that it was their faith in God and prayers for protection that helped them to remain negative.**

Some men felt embarrassed to undertake domestic responsibilities designated for women outside the home where neighbors could see them. They would therefore undertake such responsibilities at odd hours to avoid being seen by neighbors. - What are those odd hours? Provide more context from the data. I also do not think the quotation that follows would have “odd hours” mentioned in the local language. Is that the case? Or were there specific times mentioned.

**Response: Thank you for this comment. We have clarified the odd hours to mean early morning hours before the neighbours could see them**

I notice inconsistences in quotes that have Interview and respondent included. In one cases you provide these in full, and in another you provide initials. Pages 20, 21 and 24

**Response: These have been reviewed and we have ensured consistency in the presentation**

The second quotation under Finding support to manage household needs and businesses does not relate to the synthesized text. On page 22

**Response: The quote has been deleted.**

Under strengths and limitations its mentioned that: The strength of this study lies in collecting information from caregivers from varied residential areas which broadened the scope of responses.. – But, the study findings do not provide that distinction. This would cease to be a strength if the results do not provide this distinction. They are all bundled up currently.

**Response: The results have been revised to highlight the differences, as such, we have maintained the point of drawing from various settings as a strength**
