# Supplementary material for: “Why take the patient back home?”: Exploring the lived experiences of caregivers of COVID-19-infected individuals in Blantyre, Malawi
Source: PLOS Glob Public Health. 2023 Sep 27;3(9):e0001601. doi: 10.1371/journal.pgph.0001601 (PMC10529612; doi:10.1371/journal.pgph.0001601)
Supplement: Attachment — Submitted filename: Global Health Review May 27_23(1).docx [file pgph.0001601.s005.docx]

**Overall impression**

In this research manuscript, the authors describe the experience of caregivers of COVID-19 patients in Blantyre Malawi. The authors used existing theory to organize qualitative results and found the socioeconomic status of the household strongly influenced the experience of the caregivers. Caregivers’ experience involved carefully balancing many other issues such as caring for the patient, protecting their own health, following isolation precautions, getting support from outside, and looking to the future for hope. The conclusion of the study is that caregivers require clear, reliable health information and many households also require financial support. The conclusion of the study is credible, however further improvements to the manuscript are needed so the methods and results are adequately described for the reader.

**Major Feedback**

1. The overall impact of the manuscript would be improved if the writing is rephrased in the active voice and condensed.

Examples

- Discussion section - First 8 lines of the last paragraph on page 25 are difficult to understand. Suggestion…. “ Authors of previous studies demonstrated that families who followed recommended isolation measures for COVID-19 experienced increased fear, increased stress related to financial demands and health concerns, and decreased social support (23-26). Our findings are consistent with previous studies and further demonstrate how following isolation is more difficult in crowded settings…
- Discussion section - second paragraph of page 26, consider instead…. . “The results of our study also illustrate caregivers’ need for credible information on home management of COVID-19. This finding is supported by earlier studies on this topic. Clear information on the home management of COVID-19 would help caregivers by…

2. In the Methods section (sub-section: analysis), the authors need to name the specific theory used and the authors of theory. The authors also need to more explicitly describe how the theory was used to deductively analyze the results (describe the deductive coding scheme and how it was developed)
3. The authors should provide a different explanation to justify the adequacy of the sample size. The reference to Guest does not strengthen the justification that saturation was achieved. The manuscript would be stronger if this reference was removed. As cite, this explanation is inconsistent with other explanations of data saturation in qualitative research. Instead the authors could provide more description of how they knew they had collected enough data. (i.e., was the data analyzed during collection, how did the authors know they would be able to provide a full description with substantiated ideas)
4. Some of the results, particularly the results on economics (theme 1) and fear related to covid 19 death rates are written in the words of the authors and sound as though they contain author interpretation. This undermines credibility of findings. The authors should rephrase many of these statements using evidence from the caregivers/transcripts. For example, they could rephrase as “the Caregivers stated concerns about “ or “Caregivers who lived in ___ neigbourhoods often described____, where as caregivers who lived in _____ neigbourhoods did not report these experiences.

Examples:

- page 11, 1^st^ paragraph, 5^th^ line, “participants who regarded themselves as poor”- how do the authors know they regarded themselves this way? Were participants asked this question? How did they respond? Did the participants use the word poor? Perhaps you could say that participants who expressed economic concerns also described difficulties caring for their patient at home, whereas those who did not…
- page 11, first line- last paragraph, “economic challenges were more pronounced”- again how did the researchers know they were more pronounced? Did these caregivers mention money problems more often? Were the types of examples they were providing more dire?
  - page 12, 2^nd^ last line- “caregivers from all residential areas were committed”- instead say how you learned they were committed; did the caregivers express deep concern for patient? Did the caregivers describe sacrificing other things to support the patient?

5. In the methods section (sub-section: study setting section) more information is needed to describe how the caregivers’ neigbourhood of residence (and possibly occupation) were used as proxy for socio-economic status. This information is mentioned in the limitations section, but should be introduced earlier and with additional description if possible.

**Minor feedback and section by section results**

1. enrollment is spelled inconsistently throughout the manuscript
2. Abstract- need to name the sampling strategy (purposive) and the method of analysis
3. Abstract- As per major feedback, the results in the abstract sound as though they are written in the words of the authors and would be more credible in terms such as “caregivers described feelings of…” or “caregivers expressed difficulty in…..”
4. Abstract- Conclusion- the first sentence is repetitive from the 1^st^ sentence of results and could be cut
5. Introduction- end of 2^nd^ parag, page 3 and bottom of page 3, would be more clear to the reader if these statements were rephrased in terms of what were the government recommendations were for home caregiving and isolation at the time
6. Introduction - bottom of page 4- would manuals actually be the most helpful things for the participants in this situation? Can more practical uses of the data be described (as in the conclusions section of the manuscript (e.g. infographics, verbal information from Community Health Workers, financial support programs)?
7. Methods-Page 5 – setting- 1^st^ parag, lines 4-7. Could condense the manuscript if you cut sentences “The city is not well planned….. economic sustenance” as this information is repeated later in paragraph.
8. Methods-bottom of page 5- can you draw on other research or government census data to say something about the income level (relative to the avg. income of Malawi) of people who live in rural, urban low density, and urban high density neighbourhoods. Perhaps describe the types of employment people tend to have in different neighbourhoods?
9. Methods-move lines 7-9 in 2^nd^ paragraph of page 7 to page 6 in section about saturation (as per major feedback item #3)
10. Methods- Please describe if the interviews were structured or semi structured

11. Methods (and throughout the manuscript- including figure 1). The theory used in the analysis should at minimum be cited (and perhaps also named) each time it is referred to in a new paragraph.

12. Methods-eliminate the word “openly” from line 4, bottom of page 8 – open coding is a different process whereby researchers develop the codes from the data, rather than apply pre-developed codes to the data, which I believe is not what was done in this work.

13. Methods- Remove words like correct and accurate from the first two paragraphs of page 9 as they are not consistent with the qualitative research paradigm. In the qualitative paradigm, different interpretations can be accurate and correct as long as they are explained and supported by data

14. Methods-page 9, 2^nd^ paragraph- Authors should state who actually did this; could cut second sentence-not necessary as the process was previously described.

15. Methods- move last sentence of ethics section to early in the analysis section

16. Results- The authors could consider using the term dimensions or elements to describe “themes”. Dimensions or elements may be more appropriate, since thematic analysis was not used.

17. Results- In the last sentence of page 9- “twenty four caregivers were employed” is this formal employment or self-employment? Is there anything that could be added here socioeconomic status of the participants? It would help to substantiate the main theme.

18. Results – In the first paragraph of page 10- Overall try to shorten this paragraph, but also include a sentence describing Figure 2. The structure of the results is quite complex and a bit more explanation would be helpful.

19. Results - Instead of numbering the themes 1,2,3,4, it would be more helpful to call the economic dimension the “underpinning” dimension and commitment, expectation management, and caregiving role, the interconnected dimensions of the overall lived experience.

20. Results On page 10, line 5- As per previous feedback, this information should be in analysis section and described more fully there

21. Table 1- occupation row—More information on each category would help readers from outside Malawi understand the socioeconomic background of the caregivers? For example—does employed mean formal employment, business- mean self employment/farming/ informal work?

22. Results - Page 12- first sentence of theme 2- include this information in the theoretical framework section.
23. Discussion (Strengths and Limitations) **-** Top of page 27- first sentence consider rephrasing as “, however the research assistants mitigated this limitation by probing for more information…”
24. Discussion - As per previous feedback - Information that researchers used neighbourhood as proxy for economic status should be placed earlier in the manuscript and explained so that readers understand this when they are reading the results.
