# Supplementary material for: “Why take the patient back home?”: Exploring the lived experiences of caregivers of COVID-19-infected individuals in Blantyre, Malawi
Source: PLOS Glob Public Health. 2023 Sep 27;3(9):e0001601. doi: 10.1371/journal.pgph.0001601 (PMC10529612; doi:10.1371/journal.pgph.0001601)
Supplement: Attachment — Submitted filename: Letter of Response, August 2023.docx [file pgph.0001601.s006.docx]

**Dear Editors and Reviewers,**

We appreciate the review that was made to our manuscript. It has strengthened the message we want to communicate with the readers. Please find responses to the queries raised below:

**Journal Requirements:**

In the ethics statement in the Methods, you have specified that verbal consent was obtained. Please provide additional details regarding how this consent was documented and witnessed, and state whether this was approved by the IRB.

Response: This was provided for in the last revision and it reads as follows:

Ethics approval was obtained from the Kamuzu University Research Ethics Committee, College of Medicine Research and Ethics Committee (CoMREC) (P.05/20/3046). The Director of Health and Social Services for Blantyre granted the research approval to conduct the study in Blantyre.  All participants provided verbal consent cognizant that these were telephonic interviews. To limit physical contact and prevent the spread of COVID-19, a research assistant explained to a prospective volunteer that agreement would be obtained through telephone. A research assistant also stated that the potential participant will be given the opportunity to have the IRB-approved consent form read out to them before deciding whether or not to participate in the study. This procedure was not observed because it was difficult to follow such procedures over the phone. The consent form was read to a participant after the previously stated explanation, and he or she orally accepted to take part in the study. The audio recording shows the reading of it out loud and the decision-making process. The process of verbal consenting was approved by the Ethics Committee as a measure of preventing the spread of COVID-19 infection

All references have been checked and are correct.

**Reviewer 1**

**Major Feedback**

1. The overall impact of the manuscript would be improved if the writing is rephrased in the active voice and condensed.

Examples

- Discussion section - First 8 lines of the last paragraph on page 25 are difficult to understand. Suggestion…. “ Authors of previous studies demonstrated that families who followed recommended isolation measures for COVID-19 experienced increased fear, increased stress related to financial demands and health concerns, and decreased social support (23-26). Our findings are consistent with previous studies and further demonstrate how following isolation is more difficult in crowded settings…
- Discussion section - second paragraph of page 26, consider instead…. . “The results of our study also illustrate caregivers’ need for credible information on home management of COVID-19. This finding is supported by earlier studies on this topic. Clear information on the home management of COVID-19 would help caregivers by…

Response: We have revised the discussion section to make it easier to read and follow.

2. In the Methods section (sub-section: analysis), the authors need to name the specific theory used and the authors of theory. The authors also need to more explicitly describe how the theory was used to deductively analyze the results (describe the deductive coding scheme and how it was developed)

Response: The author has been included. Kindly note that the name of the theory was already in the manuscript “theory of caregiving dynamics”. The revised sentence reads as follows:

The qualitative data on the lived experiences of the caregivers were analysed and categorised following a deductive approach and guided by the theory of caregiving dynamics (Figure 1) [15]. This is a nursing and was developed by Loretta Williams. It has three constructs as follows: commitment, expectation management, and role negotiation.

We have added more depth to the analysis section as follows:

The codebook was deductively developed following the construct of the theory of caregiving dynamics to initially code the transcripts in terms of the caregivers’ experience, the challenges and measures that were taken to solve the challenges, the actions the played in rendering care such as being available, sourcing support from others. Inductively, we coded the data based on what was found for instance economic aspects are not highlighted in the theory of caregiving dynamics but were more prominent in the data. Once the codebook was ready, all data was coded by an independent and experienced data coder, a social scientist who is conversant with NVivo. The data coder was oriented to the codebook and held iterative meetings with the researchers to discuss the inclusion of more codes in the codebook or merge overlapping codes. The coded data were reviewed for completion and accuracy by MC and ALNM. Thereafter, the researchers reviewed the data set for patterns including similarities and differences across the codes, and grouped all related data under overarching themes. The themes were named after main constructs of commitment, expectation management, and role negotiation. The data that was organized under the predetermined themes and the data that could not fit under them had their theme developed from the data for instance the economic-related theme was inductively realised from the data.

3. The authors should provide a different explanation to justify the adequacy of the sample size. The reference to Guest does not strengthen the justification that saturation was achieved. The manuscript would be stronger if this reference was removed. As cite, this explanation is inconsistent with other explanations of data saturation in qualitative research. Instead the authors could provide more description of how they knew they had collected enough data. (i.e., was the data analyzed during collection, how did the authors know they would be able to provide a full description with substantiated ideas)

Response: We have included a sentence that sheds more light on saturation which ultimately guided the sample size. The revised section reads as follows:

Data analysis commenced during data analysis with an aim of ascertaining saturation of ideas to further guide the sample size. Data saturation [12] was realised when there were no more new ideas from the participants when we got to the 34^th^ interview. However, we conducted three more interviews to confirm saturation and maximise the number of caregivers from the rural areas who were initially few in the study.

Further to this- our earlier submission had the following text which shed more on the sample size, beyond the assertions by Guest

Thirty-seven caregivers were deemed adequate because Guest et.al., [11] argue that by the 12th interview, one would have captured 97% of the required information. We further observed similar themes in subsequent transcripts of the urban-based caregivers with few transcripts for rural based-caregivers which was also in keeping with the pattern of the pandemic, with the urban areas worst affected than rural settings. We, therefore, increased the sample size for the rural-based caregivers to check if they would be different themes in the transcripts for the rural-based caregivers and to maximise the number of rural-based caregivers as they were few. We noticed no new information as we progressed with the additional interviews suggesting we had reached data saturation [12].

4. Some of the results, particularly the results on economics (theme 1) and fear related to covid 19 death rates are written in the words of the authors and sound as though they contain author interpretation. This undermines credibility of findings. The authors should rephrase many of these statements using evidence from the caregivers/transcripts. For example, they could rephrase as “the Caregivers stated concerns about “ or “Caregivers who lived in ___ neigbourhoods often described____, where as caregivers who lived in _____ neigbourhoods did not report these experiences.

Examples:

- page 11, 1^st^ paragraph, 5^th^ line, “participants who regarded themselves as poor”- how do the authors know they regarded themselves this way? Were participants asked this question? How did they respond? Did the participants use the word poor? Perhaps you could say that participants who expressed economic concerns also described difficulties caring for their patient at home, whereas those who did not…
- page 11, first line- last paragraph, “economic challenges were more pronounced”- again how did the researchers know they were more pronounced? Did these caregivers mention money problems more often? Were the types of examples they were providing more dire?
  - page 12, 2^nd^ last line- “caregivers from all residential areas were committed”- instead say how you learned they were committed; did the caregivers express deep concern for patient? Did the caregivers describe sacrificing other things to support the patient?

Response: Thanks for this comment and insight. The results section has been revised to reflect that it was the participants’ narratives that are reported and not the researchers’ interpretation.

5. In the methods section (sub-section: study setting section) more information is needed to describe how the caregivers’ neigbourhood of residence (and possibly occupation) were used as proxy for socio-economic status. This information is mentioned in the limitations section, but should be introduced earlier and with additional description if possible.

Response: This has been stated as follows:

The variation in settings was deemed as a proxy to different economic status as well because it offered the researchers an opportunity to recruit participants that had different socioeconomic ranking based on area of residence.

**Minor feedback and section by section results**

1. enrollment is spelled inconsistently throughout the manuscript

Response: Thanks for this. It has been revised to be consistent.

2. Abstract- need to name the sampling strategy (purposive) and the method of analysis

Response: We have added the sampling strategy and method of analysis

3. Abstract- As per major feedback, the results in the abstract sound as though they are written in the words of the authors and would be more credible in terms such as “	caregivers described feelings of…” or “caregivers expressed difficulty in…..”

Response: This has been revised and the section now reads.

Caregivers stated that their economic status of a family largely influenced the caregiving roles and abilities because it determined the resources that a household could access or not. Caregivers expressed being committed to their role despite being ill-prepared to manage a COVID-19 patient at home in addition to fears about the contagious nature of COVID-19. They prioritised their patients’ health by ensuring that they were present to offer nutritional and medical treatment. Caregivers highlighted challenges faced in the implementation of preventive measures challenging because of financial limitations and cultural factors. They further expressed fear resulting from the increase in death rates and the lack of proper information challenged their expectation of having their patients healed. Caregivers stated that they managed their role by sharing responsibilities, getting better at their role with time, and also getting support from religious institutions and social networks.

4. Abstract- Conclusion- the first sentence is repetitive from the 1^st^ sentence of results and could be cut

Response: The first sentence has been deleted

5. Introduction- end of 2^nd^ parag, page 3 and bottom of page 3, would be more clear to the reader if these statements were rephrased in terms of what were the government recommendations were for home caregiving and isolation at the time

Response: These have been revised to reflect that the measures listed are done as per Government recommendations and guidelines .

6. Introduction - bottom of page 4- would manuals actually be the most helpful things for the participants in this situation? Can more practical uses of the data be described (as in the conclusions section of the manuscript (e.g. infographics, verbal information from Community Health Workers, financial support programs)?

Response: The word manuals has been replaced with information bulletins and leaflets

7. Methods-Page 5 – setting- 1^st^ parag, lines 4-7. Could condense the manuscript if you cut sentences “The city is not well planned….. economic sustenance” as this information is repeated later in paragraph.

Response: The sentence has been deleted

8. Methods-bottom of page 5- can you draw on other research or government census data to say something about the income level (relative to the avg. income of Malawi) of people who live in rural, urban low density, and urban high density neighbourhoods. Perhaps describe the types of employment people tend to have in different neighbourhoods?

Response: Unfortunately, that was the best we could find. We searched through the National statistical Office for such information but could not get that information.

9. Methods-move lines 7-9 in 2^nd^ paragraph of page 7 to page 6 in section about saturation (as per major feedback item #3)

Response: We have retained the information because it justifies the selection of the setting- we have also beefed up the information on saturation.

10. Methods- Please describe if the interviews were structured or semi-structured

Response; They were semi-structured and it is now specified in the section

11. Methods (and throughout the manuscript- including figure 1). The theory used in the analysis should at minimum be cited (and perhaps also named) each time it is referred to in a new paragraph.

Response: The previous review asked for inclusion of a section on the theory as below and we have also cited or mentioned the theory in the manuscript:

The qualitative data on the lived experiences of the caregivers were analysed and categorised following a deductive approach and guided by the theory of caregiving dynamics (Figure 1) [15]. This is a nursing and was developed by Loretta Williams. It has three constructs as follows: commitment, expectation management, and role negotiation. Commitment entails that a caregiver provides care to the patient irrespective of the risks, challenges, and lack of self-prioritisation that comes with the role. Expectation management states that a caregiver and a patient have expectations such as worsening or a better prognosis of the condition of the patient and includes the roles that both a caregiver and patient have to fulfill. Expectation management consists of envisioning tomorrow, getting back to normal, taking one day at a time, gauging behavior, and reconciling treatment twists and turns.  Role negotiation entails a caregiver pushing and getting grips with the caring responsibility, sharing responsibilities, attending to or listening to the patient’s voice, and vigilant bridging through negotiations with the health system in the care of the patient.

12. Methods-eliminate the word “openly” from line 4, bottom of page 8 – open coding is a different process whereby researchers develop the codes from the data, rather than apply pre-developed codes to the data, which I believe is not what was done in this work.

Response: The word openly has been removed

13. Methods- Remove words like correct and accurate from the first two paragraphs of page 9 as they are not consistent with the qualitative research paradigm. In the qualitative paradigm, different interpretations can be accurate and correct as long as they are explained and supported by data

Response: The word correct has been deleted. In the most recent version, the word accurate was not appearing.

14. Methods-page 9, 2^nd^ paragraph- Authors should state who actually did this; could cut second sentence-not necessary as the process was previously described.

Response: This is not clear, could the reviewer be more explicit of the “deed”. With the many versions and changes the trail is not very clear.

15. Methods- move last sentence of ethics section to early in the analysis section

Response: This is done as suggested.

16. Results- The authors could consider using the term dimensions or elements to describe “themes”. Dimensions or elements may be more appropriate, since thematic analysis was not used.

Response: may the reviewer be more explicit by stating an example to illustrate the change

17. Results- In the last sentence of page 9- “twenty four caregivers were employed” is this formal employment or self-employment? Is there anything that could be added here socioeconomic status of the participants? It would help to substantiate the main theme.

Response: Unfortunately we did not collect more information extra on socio-economic status hence acknowledging it as a limitation. This included both formal and informal employment.

18. Results – In the first paragraph of page 10- Overall try to shorten this paragraph, but also include a sentence describing Figure 2. The structure of the results is quite complex and a bit more explanation would be helpful.

Response: In the last review, we were advised that we needed to beef up this section to clearly illustrate the economic influence.

We have the following to explain Figure 2 more: Figure 2 demonstrates that the economic factors within a family were a platform that determined the kind of lived experiences that caregivers had while nursing a patient at home.

19. Results - Instead of numbering the themes 1,2,3,4, it would be more helpful to call the economic dimension the “underpinning” dimension and commitment, expectation management, and caregiving role, the interconnected dimensions of the overall lived experience.

Response: This has been revised as suggested

20. Results On page 10, line 5- As per previous feedback, this information should be in analysis section and described more fully there

Response- The result section has been revised after this reviewed and we wondered if this still holds merit.

21. Table 1- occupation row—More information on each category would help readers from outside Malawi understand the socioeconomic background of the caregivers? For example—does employed mean formal employment, business- mean self employment/farming/ informal work?

Response: We have qualified employed to means formal or informal while have left business outside of employment because these were carrying out businesses on a smaller scale.

22. Results - Page 12- first sentence of theme 2- include this information in the theoretical framework section.

Response: This has been revised as suggested

23. Discussion (Strengths and Limitations) **-** Top of page 27- first sentence consider rephrasing as “, however the research assistants mitigated this limitation by probing for more information…”

Response: Thank you for this suggestion, the sentence has been revised.

24. Discussion - As per previous feedback - Information that researchers used neighbourhood as proxy for economic status should be placed earlier in the manuscript and explained so that readers understand this when they are reading the results.

Response: The information about neighbourhood as proxy is placed earlier in the manuscript to provide context of the study.

We are grateful for the review made to our manuscript.

Alinane Linda Nyondo-Mipando (Corresponding Author)
