# Supplementary material for: “Why take the patient back home?”: Exploring the lived experiences of caregivers of COVID-19-infected individuals in Blantyre, Malawi
Source: PLOS Glob Public Health. 2023 Sep 27;3(9):e0001601. doi: 10.1371/journal.pgph.0001601 (PMC10529612; doi:10.1371/journal.pgph.0001601)
Supplement: Attachment — Submitted filename: PGPH-D-22-02004 R3_bedingfieldn.docx [file pgph.0001601.s007.docx]

**Why take the patient back home?: exploring the lived experiences of caregivers of**

**COVID-19 infected individuals in Blantyre, Malawi.**

**Manuscript Number: PGPH-D-22-02004R3**

The paper describes a descriptive qualitative research study of the experiences of caregivers of COVID-19 infected individuals. Analysis was guided by the Theory of Caregiving Dynamics. Authors found that caregivers’ experiences were largely shaped by financial circumstances and that caregivers felt fearful but committed to their role despite feeling ill-prepared because of a recent increase in the case fatality rate and lack of information. Some caregivers were limited in what they could do because of financial constraints but were nevertheless managed their role by being present for the patient, making sacrifices and sharing responsibilities. The authors suggest that caregivers of COVID-19 infected individuals require more information, and some require additional financial supports to manage their role.

The study supports previous findings in other settings and brings nuance from the Malawian experience. The authors have substantially improved the paper with each round of feedback, and I congratulate on their hard work.

I have no major feedback to offer and feel that the study could be published following the completion of these minor revisions.

Only minor items remain which are as follows:

1. The paper requires a general edit as additional punctuation, extra punctuation, missing words, random letters, et cetera are present throughout.
2. Abstract - the theoretical framework for the study should be identified in the methods section.
3. Introduction section, 2^nd^ paragraph, middle section. Rephrase sentence starting with *Malawi recommends…*  to read *Malawi National guidelines state that….*
4. Data collection- 2^nd^ paragraph, middle section. Rephrase sentence starting with *Data analysis began during data analysis….* This sentence is difficult to follow, but could be possibly be stated as *Data analysis began during data collection with the aim of determining saturation and sufficiency of the size of the sample*
5. Ethic approval section- 1^st^ paragraph- please explain the informed consent process which was ultimately completed with the participants more clearly. What exactly was explained to the potential participants? From what is written, it is not clear if it was it a summary of the consent form or the entire consent form.
6. Discussion section- 2^nd^ paragraph- Suggest deleting the sentence which begins with *We assert that with careful preparation and assessment of ultra-poor families… .* This assertion is beyond the scope of the data. The authors could choose to keep the sentence, but they would need to support it with additional evidence (budgetary or health economics evidence?) for it to be credible.
7. Discussion section- 2^nd^ paragraph- Rephrase sentence which begins with *Notably, industrialized nations…* Do perhaps the author mean to say “*Industrialized nations have supported households affected by COVID-19 with….*
8. Discussion section- 5^th^ paragraph- The authors generalize beyond their data with recommendations on spirituality. Perhaps a more modest claim, could be that religious support may help many caregivers in Malawi (or in the Blantyre area) .
